# Late Complications of TAVR: Endocarditis and Dissection Managed With Strategic Surgery

**DOI:** 10.1016/j.jaccas.2025.105586

**Published:** 2025-10-29

**Authors:** Yuika Kameda, Mayu Nishida, Mio Kasai, Fumiaki Yashima, Mitsuharu Mori, Kenichi Hashizume

**Affiliations:** a Department of Cardiovascular Surgery, Saiseikai Utsunomiya Hospital, Tochigi, Japan; b Department of Cardiology, Saiseikai Utsunomiya Hospital, Tochigi, Japan; c Department of Cardiovascular Surgery, Keio University School of Medicine, Tokyo, Japan

**Keywords:** aortic valve, dissection, endocarditis, valve replacement

## Abstract

**Background:**

Prosthetic valve endocarditis (PVE) and aortic dissection are rare but severe complications of transcatheter aortic valve replacement (TAVR), with no standardized surgical approach.

**Case Summary:**

A 74-year-old woman developed PVE 6 months after TAVR with an Evolut PRO+ valve (Medtronic). Echocardiography revealed valve vegetation and perivalvular leakage, and computed tomography showed localized ascending aortic dissection. Emergency surgery involved careful removal of the Evolut PRO+ using sutureless aortic valve replacement and ascending aorta replacement.

**Discussion:**

Successful management of TAVR-related PVE with aortic dissection in high-risk patients requires a clear understanding of prosthetic valve characteristics and tailored surgical planning. Techniques such as cold-water explantation and sutureless aortic valve replacement can be particularly useful in minimizing operative risk.

**Take-Home Messages:**

This case highlights a reproducible strategy for complex TAVR complications. Cold-water explantation and the snare technique allows safe valve removal, while sutureless aortic valve replacement shortens procedural time, offering practical advantages in high-risk patients.

## Visual Summary

**Figure:**
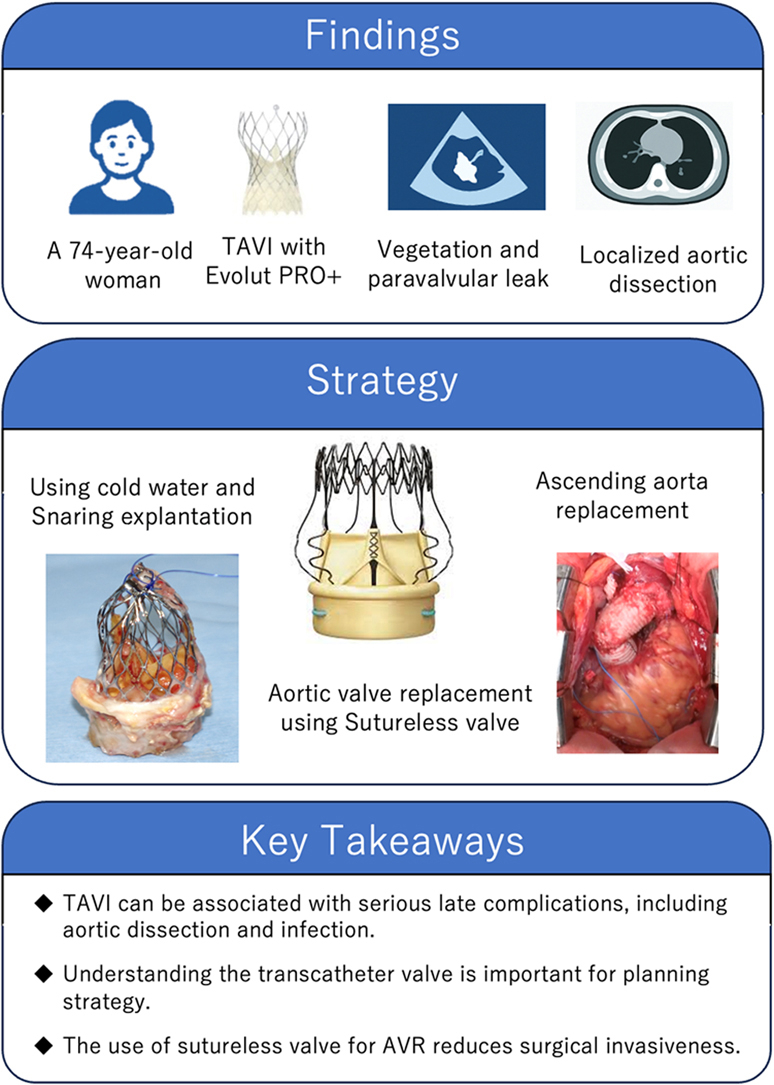

## History of Presentation

A 74-year-old woman presented with generalized weakness, a fever over 38 °C for 2 weeks, fatigue, and chills. At presentation, she was hemodynamically stable, with a blood pressure of 126/70 mm Hg, heart rate of 73 beats/min, and oxygen saturation of 98%. General and systematic examination revealed no acute abnormalities. Her body mass index was 23.1.

**Take-Home Messages**

• This case highlights a reproducible strategy for complex TAVR complications.
• Cold-water explantation and the snare 2technique allows safe valve removal, while sutureless aortic valve replacement shortens procedural time, offering practical advantages in high-risk patients.

## Medical History

Six months earlier, the patient had undergone transcatheter aortic valve replacement (TAVR) with a 26-mm Evolut PRO+ valve (Medtronic) for severe aortic stenosis. She did not report chest pain at the time of the index TAVR procedure or in the subsequent months. In addition, she had been taking steroids for nephrotic syndrome for several years. She was also suspected of having colorectal cancer and was scheduled for further examination.

## Differential Diagnosis

Given the patient's long-term use of corticosteroids, an immunocompromised state predisposing her to potential infections, including pneumonia and urinary tract infections, was considered. Additionally, tumor fever associated with colorectal cancer was included in the differential diagnosis. Considering the persistent fever that had lasted several weeks, prosthetic valve endocarditis (PVE) involving the TAVR valve was also a potential etiology.

## Investigations

**Figure 1:**
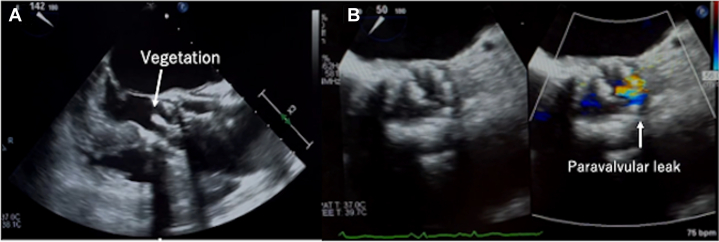
TEE of Aortic Valve Vegetation (A) TEE of aortic valve vegetation. (B) TEE with color Doppler of aortic valve paravalvular leak. TEE = transesophageal echocardiography.

**Figure 2:**
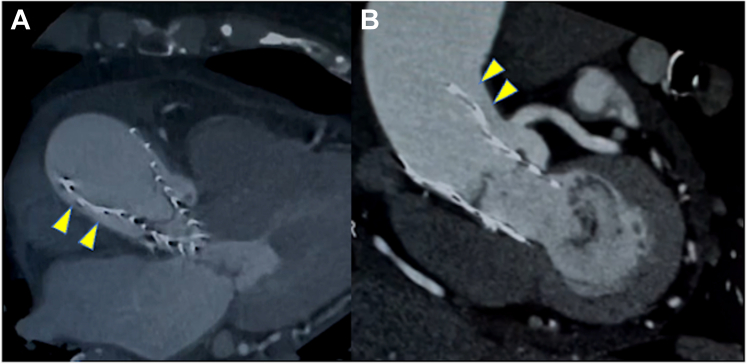
CT of Aortic Root and Ascending Aorta (A) Axial view and (B) coronal view CT revealing localized dissection of the ascending aorta at the superior border of the Evolut PRO+ stent (arrowheads). CT = computed tomography.

Blood cultures performed at the time of admission revealed *Enterococcus faecalis*. Transthoracic echocardiography (TTE) showed no obvious vegetation; however, antibiotic treatment was initiated for infective endocarditis. Transesophageal echocardiography showed a movable structure of approximately 14 mm in the leaflet on the noncoronary cusp side of the transcatheter heart valve (THV) (Figure 1A). A valvular leak was observed at the same site (Figure 1B), and the movable structure was strongly suspected to be a verruca. Perivalvular regurgitation was equivalent to mild to moderate aortic regurgitation. Magnetic resonance imaging of the head performed on the same day revealed multiple new cerebral infarcts. Additionally, contrast-enhanced cardiac computed tomography (CT) revealed localized dissection of the ascending aorta at the superior border of the Evolut PRO+ stent and a small amount of pericardial effusion (Figure 2). Notably, post-TAVR follow-up imaging, including TTE and CT performed shortly after the procedure, had shown no evidence of dissection or other acute abnormalities. A diagnosis of PVE and ascending aortic dissection after TAVR was made. Although surgery was considered high risk given her comorbidities, the PVE had verrucous mobility, indicating the need for emergency surgery.

## Management

The surgical strategy included explantation of the infected THV, followed by aortic valve replacement and ascending aortic replacement to address dissection. Given the ascending aortic dissection, direct aortic cross-clamping posed significant risk. Therefore, ascending aortic replacement was planned under deep hypothermic circulatory arrest for optimal operative control. Considering the patient's clinical condition and comorbidities, the risk of this complex procedure was deemed high. To reduce operative time and minimize surgical invasiveness, a sutureless valve was selected for the aortic valve replacement.

**Figure 3:**
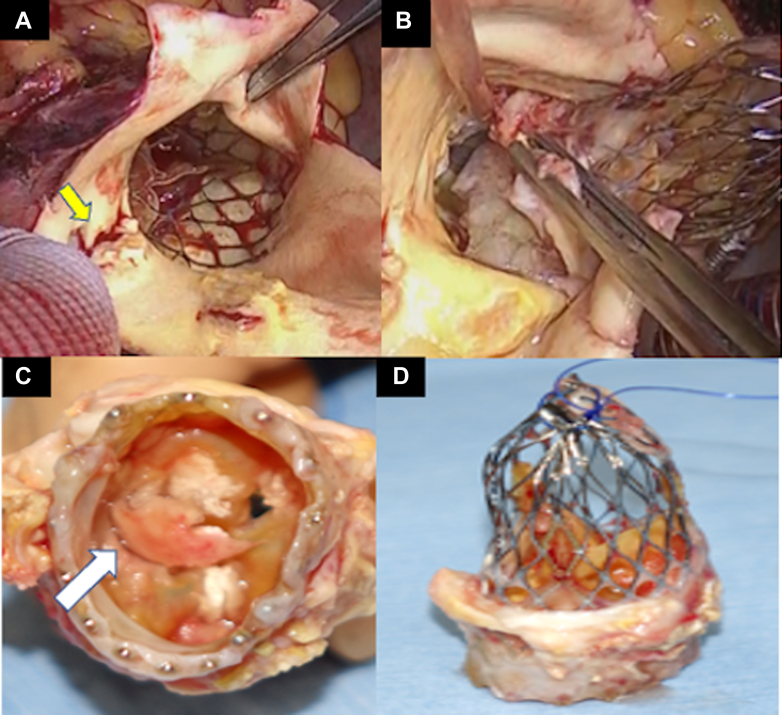
Operative Findings (A) Localized dissection (yellow arrow) on the ascending aorta at the superior border of the Evolut PRO+ stent. (B) After the Evolut PRO+ was deformed by applying ice water, it was carefully detached from the aortic intima. (C) Vegetation was adhering to the THV on the noncoronary cusp side (white arrow). (D) The Evolut PRO+ is a self-expanding prosthetic valve with a nitinol stent, which softens at low temperatures. THV = transcatheter heart valve.

Urgent surgery was performed under general anesthesia. After thoracotomy, a bloody pericardial effusion was identified. Once cardiac arrest was established with deep hypothermic circulatory arrest, the ascending aorta was incised at zone 0, where dissection was found along the superior border of the Evolut PRO+ stent Figure 3A. Inspection revealed a large vegetation adhering to the noncoronary cusp side of the THV Figure 3C. To facilitate its safe and controlled removal, a polypropylene suture was circumferentially threaded through the Evolut PRO+ stent straps. The suture was ligated, compressing the stent in a purse-string configuration Figure 3D. Ice-cold saline was applied to the Evolut PRO+ to maintain pliability, allowing careful dissection from the aortic intima Figure 3B. Although minor adhesion was observed on the noncoronary cusp side, the Evolut PRO+ was successfully removed in compressed form without significant resistance. Subsequently, heavily calcified native aortic valve leaflets were excised. Aortic valve replacement was performed using an S-size Perceval bioprosthesis (LivaNova PLC), and ascending aortic replacement was completed using a 26-mm Gelweave graft (Vascutek Terumo Inc). The procedure was completed without complications.

## Outcome and Follow-Up

The postoperative course was uneventful, with no major complications and no intracerebral hemorrhage. After completion of targeted antibiotic therapy for PVE, the patient was discharged in stable condition. At the 1-year follow-up, no recurrent infection was found, and TTE showed preserved valve function after aortic valve replacement, with no signs of dysfunction.

## Discussion

With the widespread adoption of the TAVR procedure, reports of complications requiring surgery, such as PVE and valve dysfunction, have increased. Although PVE incidence after TAVR is approximately 3%, PVE is a life-threatening complication with high mortality.^1^^,^^2^ Given the high-risk profile of patients with TAVR, medical management is often preferred, and standardized surgical approaches for TAVR-related PVE are not well established. Our patient presented with PVE complicated by ascending aorta dissection, requiring urgent surgery.

### TAVR valve explantation technique

Safe removal of the TAVR valve, particularly for self-expanding devices such as the Evolut PRO+, is a critical challenge. Owing to its self-expanding nature, the Evolut PRO+ has a nitinol frame highly integrated into the aortic wall. Various techniques for TAVR valve explantation have been reported, including snaring and lasso.^3^, ^4^, ^5^ Our approach used the cold-water technique, leveraging the unique properties of nitinol, which softens at low temperatures. This method enabled safe deformation and extraction of the Evolut PRO+ without extensive aortic wall damage. Safe removal of the TAVR valve is crucial for managing PVE, as incomplete removal or excessive wall damage can cause severe complications.

### Risk factors and mechanisms of aortic dissection after TAVR

In this case, chronic dissection localized to the ascending aorta was also observed. The incidence of aortic dissection after TAVR is reportedly 0.1% to 1.9%, making it a rare but potentially life-threatening complication.^6^ Iatrogenic aortic dissection after TAVR can occur intraoperatively or days to years after the procedure. Risk factors for early-onset dissection include anatomical characteristics such as severe calcification of the ascending aorta, atherosclerotic plaques, and aortic dilatation. Procedural factors, including guidewire manipulation and self-expanding devices, have been reported. In our case however, inflammation associated with PVE conceivably led to weakening of the aortic wall, predisposing our patient to dissection. Furthermore, the long-term use of corticosteroids may have contributed to the fragility of the vascular wall, further increasing susceptibility to dissection. This highlights the importance of recognizing that patients who have undergone TAVR, especially those with a history of steroid use or PVE, may be at increased risk of aortic dissection, necessitating vigilant monitoring and careful management.

### Role of sutureless valves in concomitant procedures

Our approach included aortic valve replacement using a sutureless valve (Perceval) and ascending aortic replacement. The sutureless valve choice was based on high surgical risk and the need to minimize operative time. Sutureless valves are advantageous in such settings because of their rapid deployment and reduced ischemic time, which are crucial for high-risk patients.

The decision to use a sutureless valve for the aortic valve replacement was based on reducing operative time and simplifying the procedure. Sutureless valves benefit high-risk patients with multiple comorbidities through rapid deployment and reduced cardiopulmonary bypass time. However, anatomical considerations must be carefully evaluated. Preoperative CT revealed a sinotubular junction (STJ) of 32 mm and an annulus diameter of 21 mm, creating an STJ-to-annulus ratio of 1.5, which exceeded the recommended limit for Perceval valves. Literature suggests that an STJ-to-annulus ratio exceeding 1.3 increases the risk of aortic regurgitation. To address this issue, we performed ascending aortic replacement using a 26-mm vascular graft with a stepwise technique, reducing the STJ diameter and ensuring compatibility with the Perceval S-size. This modification allowed successful deployment of the Perceval valve, minimizing the risk of postoperative aortic regurgitation. This case emphasizes the importance of careful preoperative planning and intraoperative flexibility in anatomically challenging cases.

### Importance of understanding valve characteristics

This case underscores the importance of understanding the specific characteristics of TAVR valves and selecting appropriate surgical techniques for their removal. The Evolut PRO+ is a self-expanding valve composed of nitinol, which is known for its temperature sensitivity. The use of the cold-water technique took advantage of this property, allowing the nitinol frame to soften and deform without causing significant aortic wall injury. This approach is safer than forceful mechanical removal, which can cause extensive damage to the aortic wall.

Additionally, our case shows the importance of anatomy in sutureless valve selection. Although sutureless valves benefit high-risk patients, their use must be evaluated based on anatomical factors such as the STJ-to-annulus ratio. Our approach showed that precise preoperative measurements and strategic adjustments can expand sutureless valve use in complex cases.

### Literature review and clinical implications

Our approach aligns with the literature supporting sutureless valves in high-risk patients owing to ease of deployment and reduced operative time. The cold-water technique has been reported as a safe method for removing nitinol-based self-expanding TAVR valves. The cold-water method is minimally traumatic and allows for controlled deformation of the valve frame.

This case shows successful management of TAVR-related PVE using the cold-water technique for valve removal and a sutureless valve for the aortic valve replacement, emphasizing the importance of valve characteristics, preoperative planning, and tailoring appropriate surgical techniques to the patient.

## Conclusions

In this patient, PVE after TAVR was complicated by chronic dissection. The cold-water and snaring techniques proved effective for safe explantation of the THV. This case highlights the efficacy of sutureless valves in high-risk procedures and emphasizes specific technical considerations when performing ascending aortic replacement with a sutureless valve. As TAVR use expands, complications requiring surgical intervention will likely increase. Understanding TAVR valve characteristics and selecting appropriate surgical techniques are essential for optimizing outcomes in PVE cases.

**Visual Summary:**
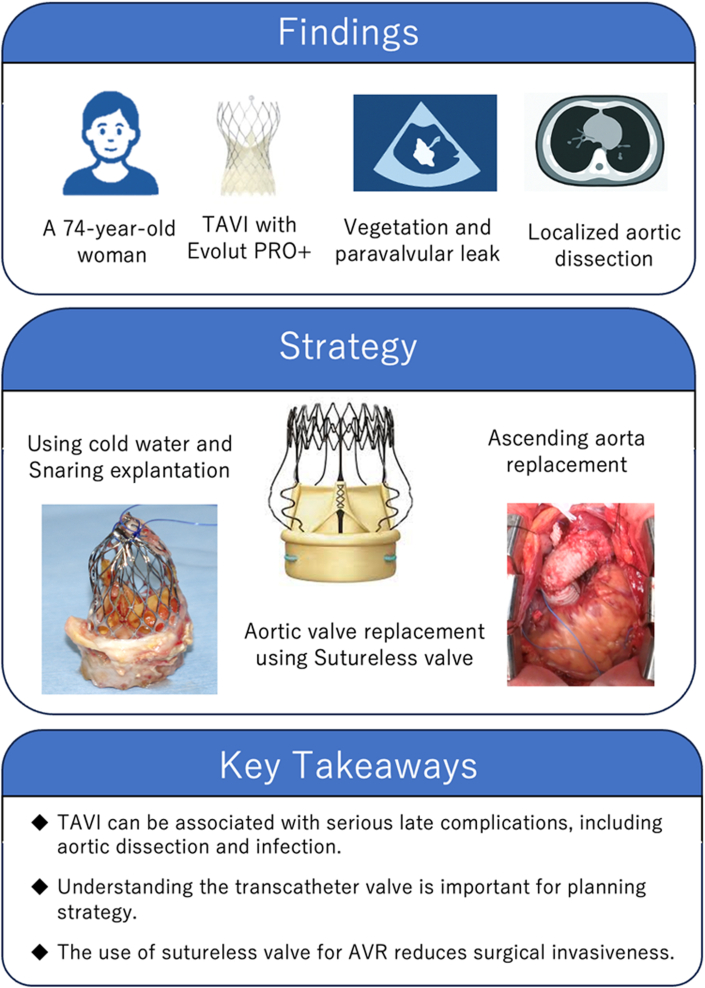
Case Presentation, Operative Strategy, and Key Takeaways AVR = aortic valve replacement; TAVI = transcatheter aortic valve implantation (TAVR).

## Funding Support and Author Disclosures

The authors have reported that they have no relationships relevant to the contents of this paper to disclose.
